# The performing arts combined: the triad of music, dance, and narrative

**DOI:** 10.3389/fpsyg.2024.1344354

**Published:** 2024-02-26

**Authors:** Steven Brown

**Affiliations:** Department of Psychology, Neuroscience & Behaviour, McMaster University, Hamilton, ON, Canada

**Keywords:** performance, music, dance, theater, narrative

## The performance triad: the arts combined

The fields devoted to the psychological study of the arts are very segregated into individual arts domains. There is very little cross-talk between the scientific fields devoted, respectively, to music, dance, theater, literature, and visual art. This situation contrasts strongly with how the arts are manifested in human cultures, in which there is a high degree of integration. One need look no further than to pop songs, opera, ballet, musical theater, cinema, music videos, performance art, and religious ceremonies to know that integration is very prevalent in the arts, despite the parallel existence of single-art formats such as instrumental music, literature, and painting. In the philosophy of art of the 18th century, the notion of a *Gesamtkunstwerk*, or total work of art, described the integrated nature of artforms such as opera and ancient Greek tragedy, where the arts combined seamlessly to create composite forms (Smith, 2007). Such an integration of the arts applies not only to the artistic realm, but to the educational applications (Winner et al., 2013) and clinical applications (Knill et al., 2004) of the arts as well.

While the absence of cross-talk between the psychological disciplines devoted to the various art domains is perhaps not surprising, there are definite drawbacks to not being able to think outside of one's own field of study. Comparison is one of the most important means of generating new knowledge. In my book *The Unification of the Arts* (Brown, 2022b), I argued that “a comparative analysis of the arts provides greater insight into each artform than is possible by looking at artforms in isolation” (p. 23). Cross-arts research is not very common in the scientific fields devoted to the psychology of the arts, despite the insights that a comparative approach could offer.

**Figure 1:**
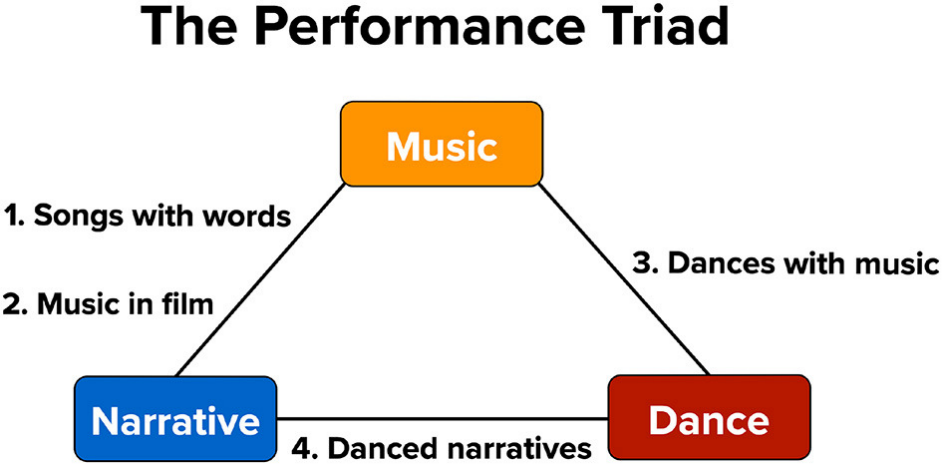
The performance triad of music, dance, and narrative. Four principal two-art couplings are shown. Danced narratives typically contain music and thus constitute a three-art coupling.

**Figure 2:**
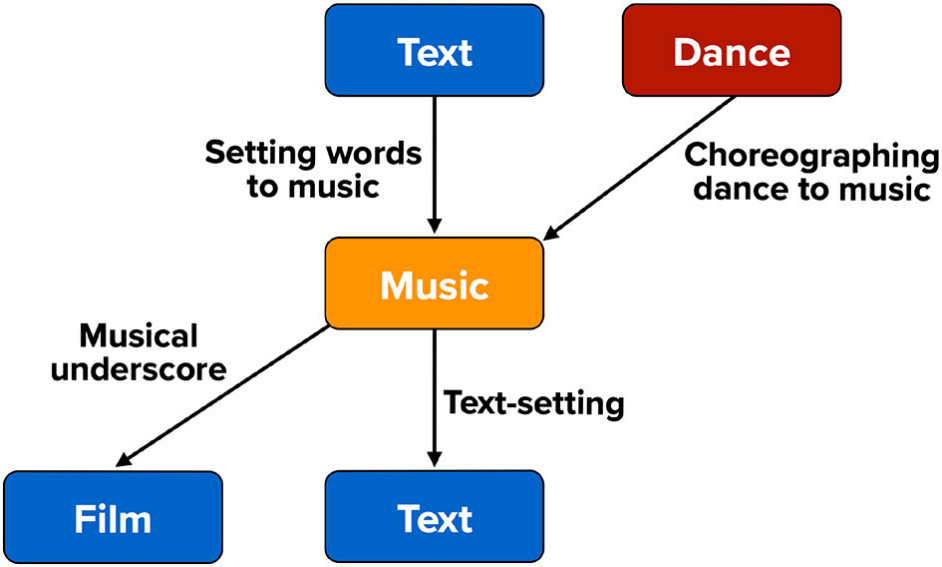
The dominant directionality in cross-arts couplings. Dance is typically choreographed to music. Musical underscore is typically composed to a produced film. The association between text and music in songs with words is bidirectional. It is as commonly achieved by creating text for already-composed music as by composing music for an existing text, the latter known as text-setting.

My aim in the current article is to shoot for the lowest-hanging fruit and to point out the most obvious couplings between branches of the arts that could be empirically studied in cross-arts research. This is shown in Figure 1 as the “performance triad” of music, dance, and narrative (Brown, 2022b). I use the word “narrative” instead of the more performance-related concept of theater since I want to include music's connection with verbal texts (e.g., poetry) in songs with words, and this can occur outside of the context of theater. My discussion of the interconnections between the artforms is in no way meant to be comprehensive. For example, I do not mention the visual arts here because of their more-remote connection to the performing arts. Instead, the aim it to highlight four areas where cross-domain research could be most easily carried out in order to get cross-arts research out of the starting gate. These areas are presented in descending order of their prevalence in the research literature: songs with words, music in film, dances with music, and danced narratives. Figure 2 shows the dominant directionality of these cross-arts couplings, since most applications are not equally bidirectional. Note that space limitations prevent me from discussing the experimental work that has already been carried out in these fields. Hence, my presentation is conceptual, establishing a theoretical framework for cross-domain research in the performing arts (see also Brown, 2018, 2019).

## Songs with words

The most ubiquitous and universal coupling between music and narrative occurs in the form of songs with words. This is found in religious chants, pop songs, folk songs, children's songs, advertisement jingles, and theatrical works like opera, among many other forms. Songs with words can be distinguished from songs that use meaningless “vocables” as their syllabic carriers (Boas, 1927), as well as from instrumental music that does not use the voice as the source of pitch.

Songs with words, in contrast to instrumental music, comprise a form of *sung speech*. They thus have the same semantic potential as speech alone. They are sung narratives, just as ballets are danced narratives (see below). However, songs with words differ from everyday speech in several key respects, including the simplicity and repetitiveness of the text, the use of poetic devices like rhyme and meter, and their ritualized context of performance (Lomax, 1968). In addition, multiple singers can coordinate their parts to create group choruses, which occurs far less frequently with speech.

### Coupling

The combination of music and speech/language in songs with words is one of the strongest examples of inter-domain coupling in the arts (Lawson, 2023). Musical pitches are associated with each and every syllable in the text. At the broader level, musical phrases are aligned with linguistic phrases. In many forms of song, music brings its use of musical scales and isometric rhythms to the text, and this very often replaces the intrinsic melodic and rhythmic features of the spoken text. The direction of coupling between music and speech/language is quite bidirectional. Music can be composed for an existing text (as was the case with Elton John and Bernie Taupin)—through a process known as text-setting—or text can be created for an existing melody (as was the case with Ira and George Gershwin). Many of the art songs of the 18th and 19th centuries were musical settings of well-known poems, hence establishing a deep cross-arts connection between music and poetry.

### Two manners of singing words

Sachs (1943) presciently proposed a distinction between two manners of singing words, what he called “melogenic” and “logogenic.” The melogenic style is melody-driven and is thus dominated by the use of musical scales and metric rhythms, as is quite common in Western culture. The logogenic style, by contrast, is word-driven, and this can be seen in the diverse means of *chanting text* across world cultures, from cantillation of the Torah to “recitativo” in opera to modern-day rapping. The logogenic style is less beholden to a musical scale and a strict rhythm. The text is the driving force for the interaction between words and music, and so the rhythm is more speech-like. Logogenic song is intermediate in style between melogenic song, on the one hand, and everyday speech, on the other, hence establishing an overall musilinguistic continuum (Brown, 2017).

## Music in film

The music that one hears in the background of feature films is called underscore. Gorbman (1987) famously referred to this music as “unheard melodies”. I would amend this description by saying that underscore consists of “unheard, but deeply felt, melodies” since the impact of the music on film viewers is very strong, even when people are completely unaware that music was present in a film that they just saw. Underscore is found not just in feature films, but in television dramas, documentaries, reality shows, and virtually all filmic forms of advertising, from short ads to informercials. Most theorists agree that—just as with music's role in songs with words and danced narratives—music functions to enhance the emotional tone of a film narrative using affective devices like scale structure, melodic contour, tempo, loudness, orchestration, and instrumental timbre. Music can even convey irony by being contrastive with the narrative scenario in a film (Ireland, 2015).

### Coupling

The predominant coupling between music and film is unidirectional (see Figure 2), with the underscore generally being composed after the film is produced. The reverse coupling is found in music videos, where a film is created to conform with a pre-composed song. Likewise, a different type of coupling with music occurs during the dance numbers in film musicals (see the next section about dance). For this section, I will focus on the more standard cinemusical relationship of musical underscore composed for a film. Compared to the strong coupling between pitches and syllables in songs with words, musical underscore provides a loose coupling between music and the overall dramatic scenario of a film. Underscore is typically composed to reflect the general emotional mood of the scene, rather than the syllables of the characters' utterances (as in songs) or the rhythmic properties of the characters' movements (as in dance). In addition, the music in films is used extensively as a cohesive device to create continuity between the many shots within a scene and between successive scenes themselves (Gorbman, 1987). A common experimental protocol in this field is to present a narratively ambiguous scene of a character and to combine it with diverse types of underscore, and then examine how viewers interpret this scene differently—for example, the character's emotions and intentions—as a function of the music played (Boltz, 2001).

## Dances with music

Many of the issues mentioned above for the coupling of songs with words apply to the choreomusical relationship between dance and music. While the vast majority of dancing occurs *with* music, it is not always done *to* music, in other words to music's metrical structure (Schröder, 2018). Instead, the music may simply serve as a background element that sets the affective tone of a dance work. This is seen quite commonly in contemporary dance. Less commonly, a musical score gets composed to an already-choreographed dance work. This is seen in certain forms of contemporary dance. It is also seen in traditional *Bharatanatyam* dancing in India, where the directionality of coupling can be quite bidirectional.

### Coupling

While the coupling between dance and music is potentially bidirectional, the unidirectional coupling of choreographing dance to music is by far the dominant directionality (Figure 2), and so I will focus my attention on this. In such instances, the metrical structure of the dance's movements is made to align with the metrical structure of the music. What this means choreographically is that large movements in the dance will align with strong beats in the music. In a waltz, the largest and most forceful movements occur on the first beat of the three-beat meter. In dance musics that have syncopated rhythms—in other words, silences on the strong beats in the meter—the dance steps do not typically abide by the syncopation, but instead maintain their adherence to the strong, stable beats in the meter, as is seen in dances like salsa and tango that have syncopated musics. Beyond meter *per se*, there is typically a prosodic matching between dance and music such that fast dance movements will occur with fast tempos in the music, and that large dance movements will occur when the amplitude of the music is loud. Finally, just as with chorusing in music, multiple interacting people can coordinate their movements in group dances, spanning from couple dances to flash mobs involving thousands of people. The study of interpersonal coordination during group dancing is a promising avenue of research (Chauvigné et al., 2019).

## Danced narratives

Of the four major couplings shown in Figure 1, the phenomenon of danced narratives has been the least studied empirically, despite the ethnographic importance of narrative forms of dance in world cultural traditions (Sachs, 1937). Danced narratives generally contain music, and so all of the points mentioned in the previous section about dances with music apply here, creating a three-way coupling between dance, narrative, and music. Danced narratives typically convey stories in a wordless manner, using body gestures alone to communicate the narrative. This absence of speech makes them different from the sung speech of songs with words. The actions in danced narratives are often pantomimic gestures that are iconic of everyday movements. Narrative forms of dance, unlike mime theater, resemble standard theatrical performances in that often employ sets, props, and costumes, including masks in many world traditions. Also, unlike mime theater, dance dramas use different dancers to portray each character, in comparison to a mime actor who performs all of the roles. While theatrical forms of dance, such as ballet, convey their narratives using dance all on its own, musical theater typically interjects dance numbers into a standard spoken drama. These numbers often contain singing in addition to dancing, although not always at the same time. Hence, musical theater is something of a total work of art. French operas before the 19th century often contained balletic dance numbers, but these later fell out of fashion.

### Coupling

The coupling between dance and theater is typically unidirectional, with the scenario coming first and the choreography striving to achieve a danced representation of the drama. The ballet version of *Romeo and Juliet* uses the same characters and tells the same story as the dramatic play, but the danced version does so wordlessly using dance gestures and the accompanying music, while the dramatic play uses speech. The exception is an artform like musical theater, where speaking actors occasionally break out into dances. They do not typically speak while dancing, although they might *sing their speech* during the songs that accompany these dances. Danced narratives establish a three-way connection in the performance triad, since such works generally incorporate music as well.

## Conclusions

The psychological study of the arts is highly siloed into individual arts domains. A key step toward achieving some level of unification of the arts is to consider the simplest level of coupling, namely two-art combinations. Starting from the triadic arrangement of music, dance, and narrative shown in Figure 1, I discussed four basic combinations of these artforms in order to establish inroads toward overcoming the canalization in the scientific study of the arts. Music couples with both speech and dance in a seamless, phrase-based manner to achieve hybrid artforms. Dramatic scenarios couple more loosely with music and dance. However, when they do, they produce three-art combinations that offer a strong level of integration of the arts, as seen in narrative ballet and film musicals.

There are a number of advantages to carrying out cross-domain research in the arts. (1) At the cultural level, the arts are highly integrated (e.g., opera, musical theater, cinema, religious ceremonies), and so we need psychological theories that embrace this integration, rather than brush it aside. (2) Along these lines, we need to understand the cognitive and neural mechanisms by which artforms are able to combine to form composite functions, such as how a poem can be set to music, or how a dance can be choreographed to music. (3) There are a number of co-evolutionary theories of the arts—such as the co-evolution of music and speech (Rousseau, 1781; Spencer, 1857; Darwin, 1871), and the co-evolution of dance and musical rhythm (Brown, 2022a)—and we need cross-domain research in order to examine the validity of such models. (4) Co-evolutionary theories of the arts are predicated on a *sharing* of processing resources between artforms, and so we would like to understand which mechanisms are shared between artforms, compared to which mechanisms are unique to each individual artform (Brown, 2022b). (5) Finally, I can simply reiterate what I said in the introductory section that a comparative approach to the arts provides greater insight into each artform than is possible by looking at artforms in isolation. We often come to understand what something is by understanding what it is not.

## Author contributions

SB: Conceptualization, Writing—original draft.
